# Educational intervention to improve secondhand smoke awareness, competency, and screening among health professions students

**DOI:** 10.1186/1940-0640-7-S1-A2

**Published:** 2012-10-09

**Authors:** Lisa Merlo, Noni Graham, Mark Gold

**Affiliations:** 1 Department of Psychiatry, University of Florida College of Medicine, Gainesville, FL, USA; 2 Departments of Psychiatry, Neuroscience, and Anesthesiology, University of Florida College of Medicine, Gainesville, FL, USA

## 

Alcohol use and tobacco smoke exposure are frequently correlated, with many individuals smoking and/or experiencing secondhand smoke (SHS) exposure while drinking (e.g., in bars, bowling alleys, or clubs). Unfortunately, most medical school curricula do not adequately address the topic of screening and brief intervention (SBI) for tobacco use, SHS exposure, and/or problematic drinking. The aim of this study was to assess the effectiveness of a three-part educational intervention for health-professions schools to address the consequences of SHS exposure and develop SBI skills in medical students. Because information regarding the effects of smoking and SHS can be effectively communicated via self-directed online lectures, we developed an online training module including a videotaped lecture series with accompanying PowerPoint slides. To help students develop diagnostic skills related to SHS and other substance exposure, 10 standardized patient cases were included for instruction and testing. Finally, a clinical-instruction DVD provided examples of how practitioners can implement motivational-interviewing strategies in behavior change counseling related to tobacco use, with or without co-occurring alcohol use. Participants in the educational intervention, compared with controls (no intervention), scored significantly higher on the SHS Competency Exam. In addition, 100% of students who received the intervention reported plans to screen for SHS exposure. This educational intervention may be a valuable addition to medical and health professional school curricula while requiring minimal faculty time and effort.

